# Short- and Long-Term Outcomes in Very Low Birth Weight Infants with Admission Hypothermia

**DOI:** 10.1371/journal.pone.0131976

**Published:** 2015-07-20

**Authors:** Hung-Yang Chang, Yi-Hsiang Sung, Shwu-Meei Wang, Hou-Ling Lung, Jui-Hsing Chang, Chyong-Hsin Hsu, Wai-Tim Jim, Ching-Hsiao Lee, Hsiao-Fang Hung

**Affiliations:** 1 Department of Pediatrics, MacKay Memorial Hospital, Hsinchu Branch, Hsinchu, Taiwan; 2 Department of Medical Technology, Jen-Teh Junior College of Medicine, Nursing and Management, Miaoli, Taiwan; 3 Department of Pediatrics, MacKay Children’s Hospital, Taipei, Taiwan; NIH, UNITED STATES

## Abstract

**Background:**

Neonatal hypothermia remains a common problem and is related to elevated morbidities and mortality. However, the long-term neurodevelopmental effects of admission hypothermia are still unknown. This study attempted to determine the short-term and long-term consequences of admission hypothermia in VLBW preterm infants.

**Study Design:**

This retrospective study measured the incidence and compared the outcomes of admission hypothermia in very low birth weight (VLBW) preterm infants in a tertiary-level neonatal intensive care unit. Infants were divided into the following groups: normothermia (36.5–37.5°C), mild hypothermia (36.0–36.4°C), moderate hypothermia (32.0–35.9°C), and severe hypothermia (< 32°C). We compared the distribution, demographic variables, short-term outcomes, and neurodevelopmental outcomes at 24 months of corrected age among groups.

**Results:**

We studied 341 infants: 79 with normothermia, 100 with mild hypothermia, 162 with moderate hypothermia, and 0 with severe hypothermia. Patients in the moderate hypothermia group had significantly lower gestational ages (28.1 wk vs. 29.7 wk, *P* < .02) and smaller birth weight (1004 g vs. 1187 g, *P* < .001) compared to patients in the normothermia group. Compared to normothermic infants, moderately hypothermic infants had significantly higher incidences of 1-min Apgar score < 7 (63.6% vs. 31.6%, *P* < .001), respiratory distress syndrome (RDS) (58.0% vs. 39.2%, *P* = .006), and mortality (18.5% vs. 5.1%, *P* = .005). Moderate hypothermia did not affect neurodevelopmental outcomes at 2 years’ corrected age. Mild hypothermia had no effect on short-term or long-term outcomes.

**Conclusions:**

Admission hypothermia was common in VLBW infants and correlated inversely with birth weight and gestational age. Although moderate hypothermia was associated with higher RDS and mortality rates, it may play a limited role among multifactorial causes of neurodevelopmental impairment.

## Introduction

Neonatal hypothermia soon after birth remains a common issue worldwide, especially in very low birth weight (VLBW) preterm infants [1]. The incidence of hypothermia upon admission to the neonatal intensive care unit (NICU) in VLBW preterm infants is 31%–78% [2,3]. Although cold stress–induced cooling may help protect the brain of asphyxiated newborns, prolonged exposure to cold should be avoided because it is associated increased risk of death [4–7]. Hypothermia also has been associated with hypoglycemia [8], hypoxia, acidosis [9], coagulation defects, chronic lung disease [10], a higher incidence of intraventricular hemorrhage [6,11], and an increased risk of late-onset sepsis in neonates [12]. Therefore, minimizing the extent of neonatal hypothermia after birth is crucial for caregivers.

Newborn infants generally experience a drop in temperature immediately after birth via evaporative, radiant, convective, and conductive heat loss [12]. The heat loss for very preterm infants in the delivery room is considerably greater than that for full-term infants [1]. A VLBW infant has many disadvantages while facing thermal stress, including the inability to produce nonshivering thermogenesis, immature skin, limited subcutaneous fat, a relatively large surface-area-to-body-mass ratio, a poorly developed response to thermal stress, and the inability to mobilize additional stabilization mechanisms. Limitations among these resources in premature infants may make them particularly vulnerable to hypothermia [13–15]. Even when caregivers follow routine thermal care guidelines, keeping preterm infants sufficiently warm immediately after birth is challenging [6,16]. Therefore, monitoring and controlling admission temperature in VLBW infants in the NICU warrants special attention.

The World Health Organisation (WHO) has classified the severity of hypothermia in the following manner: (1) cold stress or mild hypothermia = 36.0–36.4°C; (2) moderate hypothermia = 32.0–35.9°C; and (3) severe hypothermia = < 32°C [17]. However, the utility of and rationale for this scale have not been fully evaluated in preterm infants. The long-term neurodevelopmental outcomes of VLBW infants with admission hypothermia have not yet been studied. Accordingly this study attempted to evaluate the correlations of hypothermia with morbidity, mortality, and long-term neurodevelopmental outcomes in VLBW infants upon admission to NICU.

## Materials and Methods

### General

This retrospective cohort study was performed at a single, referral level III NICU of MacKay Children’s Hospital (Taipei, Taiwan). The medical charts of all inborn VLBW (birth weight <1500 g) infants from January 2007 and December 2010 were retrospectively reviewed. Infants with major congenital anomalies were excluded. All data were obtained from the database of the Taiwan Premature Infant Developmental Collaborative Study Group. The study was approved by MacKay Memorial Hospital’s Institutional Review Board (IRB number: 15MMHIS025e), with a waiver of informed consent. However, all patient records/information was anonymized and de-identified prior to analysis.

Standard thermal care was provided for all VLBW infants immediately after birth, including providing a warm delivery room at a minimum of 25°C, drying the infant, removing any wet blankets, putting the infant on a prewarmed blanket, using servo controlled radiant warmers, and, as appropriate, using preheated transport incubators. Additional interventions suggested by the American Heart Association 2010 Neonatal Resuscitation Program guidelines, such as the use of plastic bags or wraps or the use of transwarmer mattresses to keep infants warmer, were not performed during this study [18]. Our NICU was next to the delivery room and the transport time was within 5 mins. The first temperature on admission was measured by the rectal method and was recorded immediately after admission to the NICU. Admission temperature was further classified into normothermia (36.5–37.5°C), mild hypothermia (36–36.4°C), moderate hypothermia (32–35.9°C), and severe hypothermia (< 32°C) based on the WHO classifications [17]. Infants were divided into these 4 groups, and the distributions and outcomes were compared among groups.

We collected data regarding intrapartum and demographic variables. Antenatal steroids were defined as any doses of betamethasone given before delivery. Premature rupture of membranes was defined as membrane rupture > 18 h before labor. Clinical chorioamnionitis was diagnosed if the mother had fever, uterine fundal tenderness, and foul amniotic fluid. A low Apgar score was defined as an Apgar score < 7. Acidosis was defined as the pH of the first arterial blood gas < 7.2. Small for gestational age was defined as birth weight less than the 10th percentile for the gestational age. Delivery room resuscitation was defined as chest compressions with or without administration of medications.

### Short-term outcomes

The short-term outcome variables included respiratory distress syndrome (RDS) that required surfactant therapy, retinopathy of prematurity required treatment, necrotizing enterocolitis at Modified Bell’s Stage ≥ II, severe intraventricular hemorrhage (Grades III or IV), bronchopulmonary dysplasia, patent ductus arteriosus required treatment, cystic periventricular leukomalacia, culture-proven sepsis, and neonatal mortality. RDS was diagnosed by means of radiographic findings, and surfactant was delivered if ventilatory oxygen requirements were > 40%. Infants were diagnosed with sepsis if they had positive blood cultures for either bacteria or fungus. Neonatal mortality was defined as infant death before hospital discharge.

### Long-term outcomes

Neurological examinations and developmental outcomes were assessed at a corrected age of 24 months, but the protocol allowed a window of 23–25 months. The Bayley Scales of Infant Development, Second Edition (BSID-II) was the only tool used to assess developmental outcomes during the study period. The assessments were performed by a single trained psychologist. The BSID-II Mental Development Index (MDI) and the Psychomotor Development Index (PDI) scores were collected. Moderate to severe cerebral palsy was diagnosed when the child had nonprogressive motor impairment characterized by abnormal muscle tone and abnormal control of movement or posture. Neurodevelopmental Impairment (NDI) was defined as the presence of any of the following: cerebral palsy, hearing loss requiring amplification in both ears, blindness in both eyes, MDI < 70, or PDI < 70. Composite outcomes of NDI and death were compared among groups.

### Statistical analysis

Categorical data were analyzed using the standard χ^2^ test and Fisher’s exact test. Continuous data were analyzed using the independent *t*-test and the nonparametric Wilcoxon rank-sum test for between-group comparisons, where appropriate. Multivariate analyses were performed using the logistic regression model to identify the implications of hypothermia with respect to short- and long-term outcomes. Perinatal variables, including gestational age, chorioamnionitis, antenatal steroids, resuscitation, maternal education level, and sex, were included in the logistic regression models to identify the variables significantly associated with these outcomes. Adjusted odds ratios (ORs) and 95% confidence intervals (CIs) were computed for all outcomes. All statistical analyses were performed using SAS 9.2 (SAS, Cary, NC, USA). Statistical significance was defined as *P* < .05. All *P* values in this analysis were of the 2-sided type.

## Results

**Fig 1:**
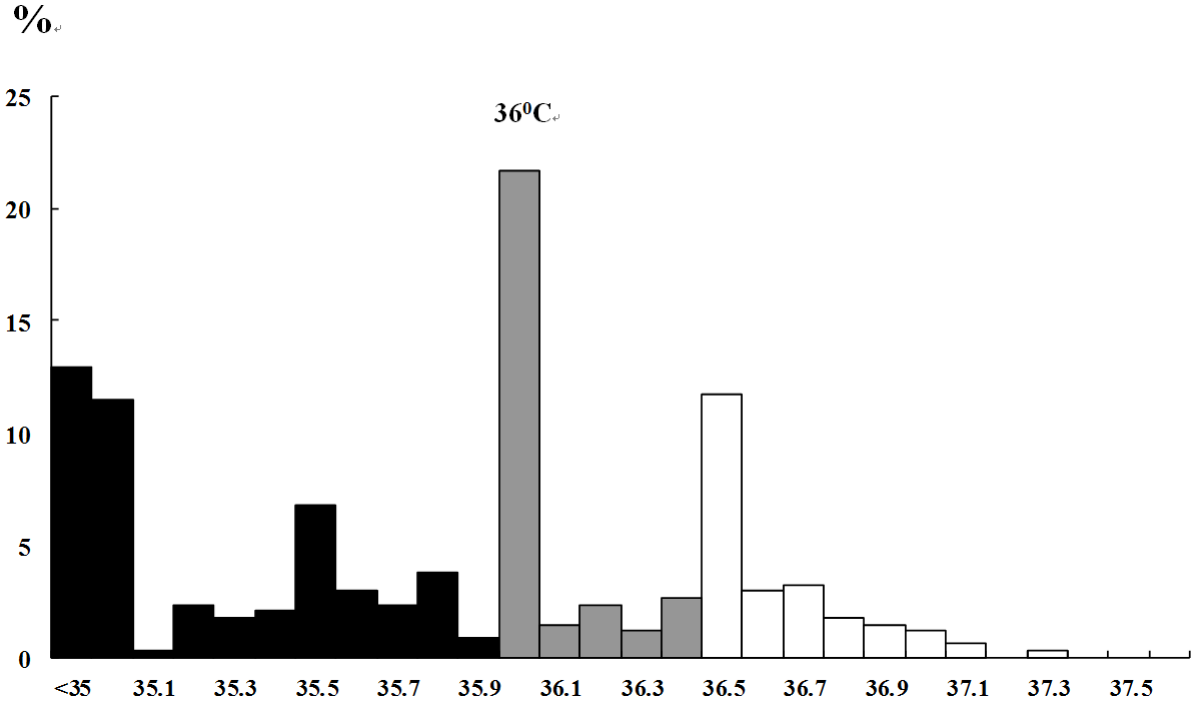
Temperature at admission to NICU (degrees centigrade).

During the 4-year study period, 341 infants met all the inclusion criteria for the study. No severely hypothermic infants were identified. There were 79 infants (23.2%) in the normothermia group, 100 infants (29.3%) in the mild hypothermia group, and 162 infants (47.5%) in the moderate hypothermia group. The frequency distribution of admission temperatures is shown in Fig 1. Hypothermia (admission temperature < 36.5°C) was found in 76.8% of the study population. The mean admission temperature was 35.6 ± 0.5°C (range, 34.0–37.3°C). The admission temperature with the highest percentage of presentations was 36.0°C (Fig 1).

The demographic data and clinical characteristics of the 3 groups are listed in Table 1. The mean gestational age was 28.9 ± 3.2 wk (range, 23–36 wk), and the mean birth weight was 1087 ± 290 g (range, 464–1498 g). The moderate hypothermia group patients had significantly lower gestational ages (28.1 wk vs 29.7 wk, *P* < .02) and smaller birth weight (1004 g vs 1187 g, *P* < .001) compared to patients in the normothermia group. Low Apgar score at 1 min (63.6% vs 31.6%, *P* < .001) was significantly higher among moderately hypothermic infants compared to normothermic infants. We found no association between groups in terms of sex, prenatal steroids, premature rupture of membranes, or resuscitation.

**Table 1:** Comparison of maternal and infant variables among groups.

	Normothermia (N = 79)	Mild hypothermia^a^ (N = 100)	Moderate hypothermia (N = 162)	*P* value^b^
Birth weight, mean ± SD (g)	1187 ± 269	1142 ± 250	1004 ± 301	< 0.001
Gestational age, mean ± SD (wk)	29.7 ± 3.1	29.5 ± 3.0	28.1 ± 3.2	< 0.02
SGA	26 (33%)	40 (40%)	61 (38%)	0.62
Male	40 (51%)	58 (58%)	84 (52%)	0.54
Cesarean section	52 (66%)	74 (74%)	117 (72%)	0.44
Antenatal steroids	65 (82%)	85 (85%)	130 (80%)	0.60
PROM > 18 h	36 (46%)	37 (37%)	53 (33%)	0.15
1-min Apgar Score < 7	25 (32%)	40 (40%)	103 (64%)	< 0.001
5-min Apgar Score < 7	6 (8%)	13 (13%)	29 (18%)	0.09
Resuscitation	11 (14%)	20 (20%)	45 (28%)	0.99
Acidosis (pH < 7.2)	10 (13%)	26 (26%)	63 (39%)	0.43

PROM, premature rupture of membrane; SGA, small for gestational age.

^a^No statistically significant *P* value: mild hypothermia group compared to normothermia group.

^b^ *P* value: moderate hypothermia group compared to normothermia group.

The total mortality rate was 13.2% (45 neonates) in this study. The mortality rate in the moderate hypothermia group was significantly higher than in the normothermia group (18.5% vs 5.1%, *P* = .05). Neonates who presented with RDS required significantly higher rates of surfactant treatment (58.0% vs 39.2%, *P* = .006) among the moderate hypothermia group compared to those in the normothermia group. These statistical differences persisted even after logistic regression analysis, which controlled for confounders. There was no association between groups regarding other neonatal short-term outcomes (Table 2).

**Table 2:** Short-term outcomes between groups.

	Normothermia (N = 79)	Mild hypothermia^a^ (N = 100)	Moderate hypothermia (N = 162)	*P* value^b^	Adjusted^c^	
					OR (95% CI)	P value
RDS required surfactant	31 (39.2%)	47 (47.0%)	94 (58.0%)	0.006	2.66 (1.27–5.58)	0.009
IVH grade III–IV	2 (2.5%)	3 (3.0%)	8 (4.9%)	0.56	1.31 (0.33–5.27)	0.43
NEC ≥ Stage II	0 (0%)	1 (1.0%)	4 (2.5%)	0.29	3.56 (0.30–41.78)	0.35
Late-onset sepsis	12 (15.2%)	10 (10.0%)	27 (16.7%)	0.54	0.85 (0.35–2.04)	0.32
PDA required treatment	21 (26.6%)	29 (29.0%)	37 (22.8%)	0.49	0.76(0.33–1.320)	0.24
ROP required therapy^d^	5 (6.6%)	7 (7.9%)	13 (9.8%)	0.63	0.85 (0.29–2.43)	0.51
Oxygen at 36 wk^d^	23 (30.7%)	24 (27.0%)	43 (32.6%)	0.81	0.83 (0.38–1.82)	0.65
cPVL^d^	1 (1.3%)	2/89 (2.2%)	5 (3.8%)	0.42	5.44 (0.43–69.62)	0.19
Mortality	4 (5.1%)	11 (11.0%)	30 (18.5%)	0.005	2.83 (0.85–8.38)	0.04

cPVL, cystic periventricular leukomalacia; IVH, intraventricular hemorrhage; NEC, necrotizing enterocolitis; PDA, patent ductus arteriosus; RDS, respiratory distress syndrome; ROP, retinopathy of prematurity.

^a^No statistically significant *P* value in the mild hypothermia group compared to the normothermia group.

^b^ *P* value in the moderate hypothermia group compared to normothermia group.

^c^Co-variates included in the logistic regression included birth weight, chorioamnionitis, antenatal steroids, resuscitation, and sex.

^d^In survivors examined. (Normothermia = 75, Mild hypothermia = 89, Moderate hypothermia = 132).

### Outcome at a corrected age of 24 months

Two hundred ninety-six infants survived to a corrected age of 24 months. Of these, 42 infants (13 in the normothermia group, 15 in the mild hypothermia group, and 14 in the moderate hypothermia group) were lost to follow-up, and thus the follow-up rate was 85.8%. Two patients with bilateral blindness did not perform BSID-II test. The infants were assessed at a mean age of 23.4 ± 1.3 months of corrected age. The mean MDI score was 87±13, 87±17, and 85±17 in the normothermia, mild hypothermia, moderate hypothermia group, respectively. The mean PDI score was 87±15, 85±14, and 80±15 in the normothermia, mild hypothermia, moderate hypothermia group, respectively. These results showed no significant differences were found between groups in terms of mean MDI or PDI scores (*P*> .05). The incidence of a composite outcome (NDI or mortality) was significantly higher in the moderate hypothermia group (48.0% vs 28.8%, *P* = .006) compared to that in the normothermia group. However, logistic regression analysis showed that after controlling for confounding variables, the significance disappeared. Admission hypothermia did not alter the rates of any other neurosensory outcomes evaluated (Table 3).

**Table 3:** Long-term outcomes between groups.

	Normothermia no./total no. (%)	Mild hypothermia^a^ no./total no. (%)	Moderate hypothermia no./total no. (%)	*P* value^b^	Adjusted^c^	
					OR (95% CI)	P value
Seizure disorder	2/62 (3.2)	3/74 (4.1)	6/118 (5.1)	0.72	1.19 (0.27–5.17)	0.59
BSID-II MDI < 70	8/61 (13.1)	10/73 (13.7)	19/118 (16.1)	0.55	1.28 (0.46–3.50)	0.41
BSID-II PDI < 70	12/61 (19.7)	13/73 (17.8)	38/118 (32.2)	0.06	0.93 (0.38–2.26)	0.15
Bilateral blindness	1/62 (1.6)	1/74 (1.4)	0/118 (0)	0.35	0.81(0.13–4.92)	0.20
Hearing impairment	1/62 (1.6)	0/74 (0)	2/118 (1.6)	1.00	0.61 (0.04–2.71)	0.14
Moderate to severe cerebral palsy	6/62 (9.7)	7/74 (9.5)	13/118 (11.2)	0.74	0.98 (0.31–3.04)	0.96
NDI^d^	15/62 (24.2)	18/74 (24.3)	41/118 (34.7)	0.12	1.08(0.48–2.45)	0.23
Death or NDI^e^	19/66 (28.8)	29/85 (34.1)	71/148 (48.0)	0.006	1.32 (0.60–2.77)	0.16

BSID-II, Bayley Scales of Infant Development, Second Edition; MDI, Mental Development Index; NDI, neurodevelopment impairment; PDI, Psychomotor Development Index.

^a^No statistically significant *P* value in the mild hypothermia group compared to the normothermia group.

^b^ *P* value in the moderate hypothermia group compared to the normothermia group.

^c^Co-variates included in the logistic regression included birth weight, chorioamnionitis, antenatal steroids, maternal education level, resuscitation, and sex.

^d^NDI is defined as any of the following: MDI < 70, PDI < 70, blindness, hearing impairment, or cerebral palsy.

^e^Data for this outcome only exclude infants who were lost to follow-up.

## Discussion

To the best of our knowledge, this was the first study to evaluate the effects of admission hypothermia on long-term neurodevelopment outcomes in VLBW preterm infants. In this cohort of VLBW preterm infants, hypothermia on admission was common in the NICU. Mild hypothermia did not affect any short-term or long-term outcomes. Moderate hypothermia was associated with lower gestational age and birth weight, as well as low Apgar score at 1 min. Although we found a significant association between the extent of reduced temperature on admission and both RDS and in-hospital mortality, the neurodevelopment and composite outcomes at 2 years’ corrected age were not affected.

The temperatures of healthy newborns have been shown to vary widely, and standard medical textbooks disagree about the lower normal limit and suggest temperatures ranging from 35.5°C to 36.5°C [19,20]. Although the WHO definitions for hypothermia are the most commonly used criteria, the definition of normothermia for the neonatal population is still unclear, and clinicians do not know whether the same definition should apply to both term and preterm infants. In the current study, the incidence of admission hypothermia was relatively higher than previously reported [2,3,5–7]. We found that birth weight was the most determinant predictor of admission hypothermia in VLBW infants. This finding was in agreement with the results of a previous study [21].

These results add weight to the urgency of initiating interventions to maintain normothermia as soon as the VLBW preterm neonate is born [16,18]. Additional and more aggressive interventions other than standard thermal management are needed in these most vulnerable populations. However, because our results revealed that mild hypothermia (36°C to 36.4°C) did not affect any short- and long-term outcomes, the clinical significance and the usefulness of the WHO definitions of hypothermia in VLBW infants requires further investigation.

Our study also showed that a low Apgar score was significantly associated with moderate hypothermia. Low Apgar scores may be related to hypothermia following increased resuscitative efforts, prolonged management time in the delivery room, or increased inherent illnesses in this population of newborns. We still do not know whether this hypothermia marks or results from a low Apgar score.

With regard to neonatal outcomes, our study found a correlation between the extent of reduced temperature on admission and RDS. Although this correlation may be an association, not a causative factor, the results are similar to those reported in a previous study [22]. Hypothermia may result in an increase in oxygen consumption, leading to hypoxemia. Hypoxemia, in turn, can cause pulmonary vasoconstriction and increased respiratory distress by impairing surfactant release and decreasing respiratory muscle efficiency. Our study also confirmed that admission hypothermia may increase mortality in VLBW infants [6,12]. Laptook et al found admission temperature was inversely related to mortality (28% increase in mortality per 1°C decrease in temperature) [12]. The chance of early neonatal death is 1.64-fold higher in infants with admission temperatures < 36°C. It remains unclear whether hypothermia at NICU admission is a cause of death or a marker of the patient’s poor condition. Because our study was an observational cohort study, the results shown here may reflect only an association. However, important associations between lower temperature at birth and subsequent morbidity and mortality suggest that temperature may either be a marker for the sickest infants or that low temperature at birth is part of the pathway to organ injury [23]. Our study revealed an increased incidence of severe intraventricular hemorrhage, necrotizing enterocolitis, and cystic periventricular leukomalacia in the moderate hypothermia group. However, these diseases occurred with a low frequency in each groups. A wide confidence interval in these outcomes and the failure to detect a significant difference may be affected by the small sample size, which indicated that the study might have been inadequately powered to detect small differences between each groups.

Our study revealed admission hypothermia did not affect neurodevelopment and composite outcomes at 2 years’ corrected age. The possible reason may be that the causes of neurodevelopment impairment are multifactorial. Hypothermia is surely one of these factors, but its causal role in long-term impairment is limited. However, a statistically insignificant trend, including a lower mean PDI scores (p = .08), a higher incidence of NDI (p = .12) and PDI < 70 (p = .06), was found in the moderate hypothermia group as compared to the normothermia group. This also can be explained by that statistical deviations may occur when sample size was small or no primary outcome was measured in this retrospective study. A well designed study with a primary outcome measured long term neurodevelopmental outcomes needs to be performed to determine the association between the risk of neurodevelopmental deficit and hypothermia.

A variety of interventions, such as uses of a stockinet or polyethylene cap, polyethylene bags or wraps, warming packs, transwarmer mattress, skin-to-skin warming, and heating respiratory gases, have been used to either decrease total heat losses or to provide external heat after birth [24]. Studies have been shown that babies < 28 wk or those weighing < 1500 g appeared to derive the greatest benefit from interventions to prevent hypothermia [24]. To optimize the possible benefits of these interventions in addition to routine care, multidisciplinary teams and multiple interventions should be implemented in patient care because heat loss is a multifactorial process. In recent studies, the use of thermoregulatory bundles has been found to be effective in improvements in admission temperatures [25–28]. However, the effects of these interventions on long-term outcomes are still unknown [24,29]. Because of the high incidence of admission hypothermia in our NICU, we currently are implementing a multi-intervention quality improvement project to improve temperature control.

An important limitation of this study is its retrospective observational design. Our data should also be interpreted with caution due to the small sample size. A large patient number may provide an ability to better detect significant differences between groups. Because no severe hypothermia patient was identified in our study, the effect of severe hypothermia on short- and long-term outcomes requires further investigation. The room temperature to which each of the VLBW infants was exposed at birth was not recorded. As noted, the most suitable environmental temperature for newborns remains controversial. In theory, the lower the baby’s birth weight, the higher the room temperature should be. A previous study suggested that ambient temperature should be in the range of 22.2–24.4°C to maintain thermal stability in VLBW infants [2]. Although WHO recommended providing a warm delivery room at a minimum of 25°C, this temperature is not always achieved in practice. Instead, delivery rooms usually are kept at a temperature that is comfortable for mothers and staff, with little consideration for the newborn. Notably, a fluctuation of 2°C in air temperature is sufficient to induce thermal stress in premature infants [28]. Other limitations of this study include the lack of data about maternal temperature at the time of or immediately proximate to delivery and the time interval between birth and arrival at the NICU. Furthermore, methods for and the accuracy of temperature measurements of newborns continue to be debated [30,31].

## Conclusions

The results of this observational cohort study demonstrate that admission hypothermia is prevalent in VLBW infants, and prompt and conscientious assessment of admission temperatures for these infants is needed. We found that the mean admission temperature decreased with decreasing birth weight and gestational age, and moderate hypothermia in VLBW infants was associated with higher RDS and mortality rates. Our results showed mild and moderate hypothermia upon admission may play a limited role in the multifactorial causes of long-term neurodevelopmental impairment. A properly designed randomized controlled trial is warranted to elucidate the relationship between low temperature at admission and important outcomes. Future studies also should focus on comparison data collected before and after any interventions in order to evaluate not only their effectiveness in improving hypothermia but also the associations between the short-term and long-term outcomes.
